# Supplementary material for: Volatile Profiles and Sensory Characteristics of Cabernet Sauvignon Dry Red Wines in the Sub-Regions of the Eastern Foothills of Ningxia Helan Mountain in China
Source: Molecules. 2022 Dec 12;27(24):8817. doi: 10.3390/molecules27248817 (PMC9782302; doi:10.3390/molecules27248817)
Supplement: Supplementary file 1 [file molecules-27-08817-s001.zip › molecules-2076026-supplementary-new version.pdf]

Supplementary material

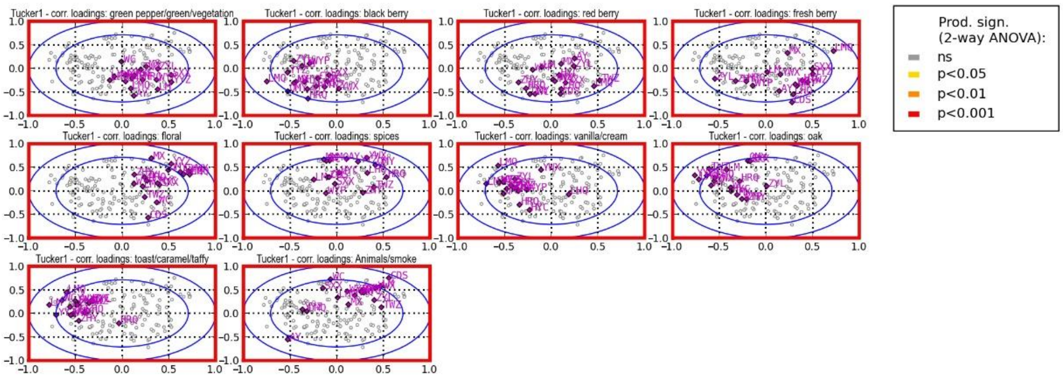

Figure S1. Two-way ANOVA analysis of typical Cabernet Sauvignon dry red wine from six sub-regions at the Eastern Foothills of Helan Mountain in Ningxia. Ten identical Tucker-1 plots with each plot highlighting one of the ten attributes used in the profiling.

Table S1. One-way ANOVA of volatile compounds of representative Cabernet Sauvignon dry red wines from five sub-regions at the Eastern Foothills of Helan Mountain in Ningxia [a]

Numbers	Compounds	Threshold [b] (μg/L)	HL	XX	YN	QTX	HSP
H1	Isobutanol	40000[26]	122348.26±34181.50a	107769.60±14608.67ab	98765.42±12627.56ab	106563.18±10730.91ab	87168.29±6809.86c
H2	Isopentanol	30000[26]	433355.93±63735.70a	406181.84±35139.14ab	371539.82±31845.02bc	370572.71±46914.12bc	322071.90±11249.16c
H4	1-Pentanol	676000[54]	59.27±7.06b	77.30±15.12a	75.82±11.93ab	64.09±4.29ab	67.12±4.43ab
H5	3-Methylpentanol	50000[55]	156.02±17.97a	141.69±25.48ab	129.42±17.46abc	121.43±19.78bc	110.52±10.83c
H6	2-Ethylhexanol	8000[56]	0.18±0.40b	1.71±2.51ab	1.35±1.48ab	2.67±0.84ab	4.41±4.13a
H7	2-Heptanol	-	3.90±0.45ab	4.61±1.40a	3.58±0.79ab	3.14±0.71c	2.83±0.44c
H10	2-Nonanol	-	0.78±0.18b	0.94±0.23b	0.88±0.14b	1.00±0.23a	0.70±0.10c
H11	1-Decanol	400[54]	2.26±0.31b	2.14±0.26b	2.16±0.16b	2.37±0.18a	1.98±0.07c
H12	Methionol	1000[54]	3524.37±1785.85a	3505.76±1482.09a	3227.93±1045.61a	2703.49±627.87a	2899.73±808.41a
H13	Benzyl alcohol	200000[54]	778.47±325.48b	969.71±795.93b	639.09±264.39b	1411.91±634.1a	466.00±68.37c
E2	Ethyl octanoate	5[26]	539.20±113.52a	358.36±116.15b	324.25±84.57b	236.07±78.18b	243.94±148.36b
E3	Ethyl decanoate	100[45]	189.92±55.83a	106.10±31.58bc	106.21±43.40bc	143.68±100.85ab	60.56±20.55c
E5	Ethyl butanoate	20[45]	177.81±43.18a	157.16±13.96ab	140.98±27.26ab	143.35±35.15ab	125.21±62.54b
E6	Ethyl hexanoate	5[54]	414.67±99.67a	347.80±28.81ab	282.77±54.57b	333.91±145.68ab	238.73±158.7b
E9	Ethyl (*E*)-3-hexenoate	-	1.12±0.14b	1.52±0.42ab	1.29±0.48b	1.91±0.13a	1.31±0.1b
E16	Hexyl acetate	1000[45]	5.00±1.40ab	3.67±0.66b	3.49±0.98b	4.31±1.00ab	5.57±1.76a
E17	Isoamyl acetate	30[54]	758.14±128.51a	485.00±110.83b	406.88±112.38b	432.00±279.32b	453.82±209.75b
E22	Methyl salicylate	-	6.57±9.36ab	7.64±10.12ab	2.92±1.10b	24.48±32.78a	3.58±2.13b
E24	Isoamyl hexanoate	-	0.97±0.13a	0.82±0.12ab	0.69±0.09bc	0.80±0.22ab	0.59±0.20c
E25	Isoamyl octanoate	125[45]	5.01±0.61a	4.43±0.22abc	4.27±0.28bc	4.80±0.96ab	4.08±0.14c
E26	Methyl octanoate	200[57]	1.41±0.14a	1.21±0.29ab	1.14±0.34ab	1.10±0.30ab	0.83±0.29b

T2	4-Terpinenol	5000[35]	0.23±0.13b	0.47±0.24ab	0.25±0.19b	0.51±0.25ab	0.73±0.51a
I1	*β*-Damascenone	0.05[26]	2.93±0.78ab	2.83±0.70ab	2.80±0.80ab	3.07±1.00a	1.85±0.29b
MP	3-Isobutyl-2-methoxypyrazine (ng/L)	2[45]	7.25±6.01a	4.92±2.40ab	2.47±2.72ab	5.93±0.88ab	1.72±2.98b
FA1	Acetic acid	200000[45]	508561.16±76162.09ab	567021.11±146916.70a	538965.25±89051.47ab	552123.78±83838.61ab	393699.48±81647b
FA2	Hexanoic acid	420[45]	1525.94±203.56ab	1405.11±271.57ab	1495.96±338.00ab	1672.92±195.83a	1143.84±257.41b
FA3	Octanoic acid	500[45]	1331.33±315.39a	1061.13±233.12ab	1109.42±214.68ab	1446.32±383.6a	867.85±129.79b
FA4	*n*-Decanoic acid	1000[45]	205.04±31.31a	162.85±14.13bc	164.65±10.74bc	186.83±16.97ab	146.44±6.22c
PA1	Vanillin	60[54]	166.72±114.06a	85.30±37.23ab	129.51±111.56ab	95.43±99.84ab	26.66±21.27a
PA2	Syringaldehyde	-	463.07±452.74b	220.12±261.80b	125.53±125.73b	439.26±118.27b	1262.65±1453.81a
PA3	Coniferaldehyde	-	27.95±5.07b	27.80±3.72b	24.66±1.59b	28.98±3.36b	93.51±99.43a
PA6	Acetosyringone	-	27.01±17.28b	33.96±18.07ab	34.99±12.92ab	64.05±44.58a	57.32±29.23ab
PA7	Acetovanillone	-	32.70±15.30a	40.13±9.43ab	40.62±9.77ab	55.58±27.39a	54.28±12.33a
VP1	Phenol	5900[45]	8.67±2.29ab	10.18±3.90a	7.97±1.67ab	9.73±1.21ab	5.85±1.39b
VP2	4-Ethylphenol	1800[45]	1.19±0.41b	1.31±0.58b	2.80±3.76ab	5.33±5.16a	1.56±0.97b
VP12	4-Vinylguaiacol	1100[45]	14.50±4.38ab	8.49±4.73b	18.39±6.3ab	49.12±62.44a	29.02±14.56ab
VP13	Cresol	-	14.61±3.92ab	15.50±4.68a	13.46±1.43ab	16.14±2.72a	10.32±0.75b
L4	*γ*-Decalactone	0.7[49]	0.56±0.13b	0.65±0.12ab	0.61±0.13b	0.64±0.09ab	0.83±0.26a
L5	*γ*-Butyrolactone	100000[58]	31808.67±9558.91a	20270.13±3389.34b	17530.92±3876.64b	20134.61±2328.28b	17088.65±4109.60b
F2	Acetylfuran	-	11.33±7.13b	17.49±13.63ab	22.51±13.98ab	12.37±6.29b	35.95±29.00a
F3	2-Furanmethanol	-	642.13±968.4b	1647.99±2069.75ab	2055.54±1816.85ab	1216.4±1127.34ab	4836.8±6558.86a
F9	Sotolon	5[26]	1.58±0.70b	2.72±0.99ab	2.82±0.69ab	2.91±1.45ab	3.45±0.72a
O2	(*E*)-2-Nonenal	-	4.92±6.88ab	8.15±4.96ab	2.74±2.62b	13.21±12.73a	3.60±1.06b

[a] The data in the table are 43 species with significant differences in the five sub-regions of the Eastern Foothills of Helan Mountain in Ningxia. The unit of IBMP is ng/L, and all the other species are μg/L. '-' represents odor threshold not found.

Table S2. Climate survey of six sub-regions at the Eastern Foothills of Helan Mountain in Ningxia in recent 30 years [a]

Sub-regions	Altitude (m)	Latitude and longitude	Annual precipitation (mm)	Annual effective accumulated temperature (°C) [b]	Sunshine duration from April to October (h)	Frost-free period (d) [c]
SZS	1117.70	39°03′,106°35′	170.88	1913.37	1600.28	186.63
HL	1106.20	38°57′,106°35′	170.967	1746.7	1681.69	187.33
XX	1110.90	38°47′,106°20′	187.697	1716.4	1569.81	180.70
YN	1113.70	38°28′,106°25′	170.533	1720.55	1604.38	184.27
QTX	1118.00	38°03′,106°08′	181.66	1711.87	1688.00	184.30
HSP	1128.80	37°98′,106°18′	183.733	1774.26	1676.69	190.37

[a] The data is from 1982 to 2011 in local meteorological. [b] The effective accumulated temperature is the cumulative sum of temperatures greater than 10°C in the grape growing season (April to October). [c] The Frost-free period means interval between the last of 0°C in spring and the first of 0°C in autumn.

Table S3. Volatile compounds of Cabernet Sauvignon dry red wine in six sub-regions at the Eastern Foothills of Helan Mountain in Ningxia

Numbers	Compounds	Class [a]	CAS	Methods	Quantitative ion (*m/z*)
MP	3-Isobutyl-2-methoxypyrazine	MP	24683-00-9	SPME-SIM	124
C6-1	1-Hexanol	C6	111-27-3	SPME	56
C6-2	(*E*)-3-Hexen-1-ol	C6	928-97-2	SPME	41
C6-3	(*Z*)-3-Hexen-1-ol	C6	928-96-1	SPME	67
C6-4	(*E*)-2-Hexen-1-ol	C6	928-95-0	SPME	57
C6-5	(*Z*)-2-Hexen-1-ol	C6	928-94-9	SPME	57
H1	Isobutanol	H	78-83-1	SPME	43
H2	Isopentanol	H	123-51-3	SPME	74
H3	1-Butanol	H	71-36-3	SPME	43
H4	1-Pentanol	H	71-41-0	SPME	88
H5	3-Methylpentanol	H	589-35-5	SPME	56
H6	2-Ethylhexanol	H	104-76-7	SPME	57
H7	2-Heptanol	H	543-49-7	SPME	45
H8	1-Octanol	H	111-87-5	SPME	56
H9	1-Octen-3-ol	H	3391-86-4	SPME	57
H10	2-Nonanol	H	628-99-9	SPME	45
H11	1-Decanol	H	112-30-1	SPME	56
H12	Methionol	H	505-10-2	SPME	106
H13	Benzyl alcohol	H	100-51-6	SPME	79
E1	Ethyl acetate	E	141-78-6	SPME	43
E2	Ethyl octanoate	E	106-32-1	SPME	88
E3	Ethyl decanoate	E	110-38-3	SPME	88
E4	Ethyl succinate	E	123-25-1	SPME	101
E5	Ethyl butanoate	E	105-54-4	SPME	71
E6	Ethyl hexanoate	E	123-66-0	SPME	88
E7	Ethyl dodecanoate	E	106-33-2	SPME	88

E8	Ethyl hexadecanoate	E	628-97-7	SPME	88
E9	Ethyl (*E*)-3-hexenoate	E	26553-46-8	SPME	41
E10	Ethyl isobutanoate	E	97-62-1	SPME	71
E11	Ethyl 3-hydroxybutyrate	E	5405-41-4	SPME	43
E12	Ethyl 2-methylbutanoate	E	7452-79-1	SPME	57
E13	Ethyl 3-methylbutanoate	E	108-64-5	SPME	88
E14	Ethyl 2-hydroxy-4-methylpentanoate	E	53530-26-0	SPME	69
E15	Isobutyl acetate	E	110-19-0	SPME	43
E16	Hexyl acetate	E	142-92-7	SPME	104
E17	Isoamyl acetate	E	123-92-2	SPME	43
E18	Phenethyl acetate	E	103-45-7	SPME	91
E19	Ethyl heptanoate	E	106-30-9	SPME	88
E20	Ethyl nonanoate	E	123-29-5	SPME	88
E21	Ethyl lactate	E	97-64-3	SPME	45
E22	Methyl salicylate	E	119-36-8	SPME	120
E23	Methyl hexanoate	E	106-70-7	SPME	56
E24	Isoamyl hexanoate	E	2198-61-0	SPME	70
E25	Isoamyl octanoate	E	2035-99-6	SPME	70
E26	Methyl octanoate	E	111-11-5	SPME	74
E27	Isoamyl lactate	E	19329-89-6	SPME	70
T1	Linalool	T	78-70-6	SPME	71
T2	4-Terpinenol	T	562-74-3	SPME	71
I1	*β*-Damascenone	I	23726-93-4	SPME	69
I2	*β*-Ionone	I	8013-90-9	SPME	177
FA1	Acetic acid	FA	64-19-7	SPME	45
FA2	Hexanoic acid	FA	142-62-1	SPME	60
FA3	Octanoic acid	FA	124-07-2	SPME	60
FA4	*n*-Decanoic acid	FA	334-48-5	SPME	60

VP1	Phenol	VP	108-95-2	LLE	94
VP2	4-Ethylphenol	VP	123-07-9	LLE	107
VP3	Guaiacol	VP	90-05-1	LLE	109
VP4	4-Ethylguaiacol	VP	2785-89-9	LLE	137
VP5	Eugenol	VP	97-53-0	LLE	164
VP6	Isoeugenol	VP	97-54-1	LLE	164
VP7	3-Ethylphenol	VP	620-17-7	LLE	107
VP8	4-Methylguaiacol	VP	93-51-6	LLE	138
VP9	4-Propylguaiacol	VP	2785-87-7	LLE	137
VP10	Syringol	VP	91-10-1	LLE	154
VP11	4-Vinylphenol	VP	2628-16-2	LLE	43
VP12	4-Vinylguaiacol	VP	7786-61-0	LLE	135
VP13	Cresol	VP	5254-12-6	LLE	107
VP14	Maltol	VP	118-71-8	LLE	126
PA1	Vanillin	PA	121-33-5	LLE	151
PA2	Syringaldehyde	PA	134-96-3	LLE	182
PA3	Coniferaldehyde	PA	458-36-6	LLE	178
PA4	Methyl vanillate	PA	3943-74-6	LLE	151
PA5	Ethyl vanillate	PA	617-05-0	LLE	151
PA6	Acetosyringone	PA	2478-38-8	LLE	181
PA7	Acetovanillone	PA	498-02-2	LLE	151
L1	Oaklactone	L	39212-23-2	LLE	99
L2	Pantolactone	L	599-04-2	LLE	71
L3	γ-Nonalactone	L	124-19-6	LLE	85
L4	γ-Decalactone	L	112-31-2	LLE	85
L5	γ-Butyrolactone	L	96-48-0	LLE	42
F1	5-Methylfurfural	F	620-02-0	LLE	110
F2	Acetylfuran	F	1192-62-7	LLE	95

F3	2-Furanmethanol	F	98-00-0	LLE	98
F4	Furaneol	F	3658-77-3	LLE	43
F5	Homofuraneol	F	451-13-8	LLE	122
F6	Furfural	F	98-01-1	LLE	96
F7	Cyclotene	F	80-71-7	LLE	112
F8	5- Hydroxymethylfurfural	F	67-47-0	LLE	97
F9	Sotolon	F	28664-35-9	LLE	83
O1	Nonanal	O	124-19-6	SPME	57
O2	(*E*)-2-Nonenal	O	18829-56-6	LLE	43
O3	Benzaldehyde	O	100-52-7	SPME	77
O4	Phenylacetaldehyde	O	122-78-1	SPME	91
O5	Styrene	O	100-42-5	SPME	104
O6	Ethyl phenylacetate	O	101-97-3	SPME	91

[a] C6, C6 alcohols; H, higher alcohols; E, esters; T, terpenoids; I, C13-norisoprenoids; MP, Methoxypyrazines; FA, Fatty acids; B, Benzenes; VP, Volatile phenols; PA, Phenolic aldehydes; L, lactones; F, furans; O, other compounds.

Table S4. The Frequency of 71 Cabernet Sauvignon dry red wines from six sub-regions at the Eastern Foothills of Helan Mountain in Ningxia [a]

Wines	Mulberry	Strawberry	Cherry	Hawthorn	Raspberry	Green pepper	Mint	Green	Grass	Violet	Jam	Spices	Sophora japonica	Apple /Pear	Banana	Peach/ Apricot
SZS-1	0.08	0.00	**0.23**	0.08	**0.20**	0.18	0.03	0.13	0.03	0.13	0.10	0.05	0.05	0.00	0.05	0.00
SZS-2	0.03	**0.20**	**0.23**	**0.23**	**0.33**	0.13	0.00	0.15	0.08	0.18	**0.35**	0.10	0.05	0.13	0.05	0.13
HL-1	0.03	0.15	**0.33**	0.05	**0.28**	0.05	0.05	0.10	0.08	0.13	**0.28**	0.15	0.08	0.10	0.10	0.13
HL-2*	0.05	0.08	**0.23**	0.05	0.13	0.18	0.03	0.13	0.03	0.13	0.15	0.18	0.05	0.05	0.03	0.15
HL-3	0.03	0.13	**0.28**	0.03	0.15	0.15	0.03	0.15	0.05	0.13	0.15	0.15	0.05	0.03	0.03	0.08
HL-4*	0.10	**0.23**	**0.38**	0.05	**0.40**	0.08	0.03	0.05	0.03	**0.23**	**0.23**	0.10	0.08	0.15	0.08	0.15
HL-5*	0.13	0.05	**0.20**	0.03	0.15	**0.28**	0.08	0.18	0.15	**0.23**	0.10	0.18	0.00	0.05	0.00	0.03
HL-6*	0.15	0.05	**0.25**	0.13	**0.28**	0.08	0.05	0.10	0.03	**0.28**	0.18	0.13	0.00	0.03	0.03	0.08
HL-7	0.05	0.03	0.18	0.05	**0.23**	0.15	0.05	0.10	0.03	0.18	0.15	0.10	0.10	0.03	0.00	0.05
HL-8	0.08	**0.23**	**0.33**	0.10	**0.33**	0.05	0.08	0.08	0.03	0.13	**0.33**	0.08	0.18	**0.23**	0.03	0.13
HL-9	0.10	0.03	0.13	0.05	0.18	**0.65**	0.05	**0.33**	**0.23**	0.08	0.05	0.18	0.03	0.03	0.03	0.03
HL-10*	0.13	0.08	**0.23**	0.08	0.10	**0.50**	0.10	**0.30**	0.18	0.10	0.13	0.18	0.03	0.08	0.00	0.10
HL-11	0.13	0.08	**0.20**	0.10	**0.30**	**0.23**	0.10	0.05	0.13	0.08	0.15	0.18	0.05	0.10	0.03	0.05
YC-1	0.10	0.08	**0.38**	**0.23**	**0.28**	**0.30**	0.05	0.05	0.08	0.15	0.18	0.13	0.03	0.05	0.00	0.05
YC-2*	0.18	0.13	**0.28**	0.18	**0.38**	0.13	0.13	0.10	0.05	**0.30**	**0.30**	0.13	0.10	0.03	0.08	0.05
YC-3*	0.10	0.15	**0.23**	0.15	**0.25**	0.18	0.10	0.15	0.10	**0.20**	0.18	**0.23**	0.00	0.05	0.08	0.15
YC-4	0.15	0.10	**0.43**	0.10	**0.30**	0.10	0.15	**0.20**	0.10	**0.28**	0.18	0.15	0.13	0.15	0.03	0.03
YC-5	0.13	0.05	**0.33**	0.08	**0.25**	**0.20**	0.13	0.15	0.08	0.15	0.08	**0.25**	0.03	0.05	0.03	0.03
YC-7*	0.15	0.13	**0.35**	0.13	**0.28**	0.15	0.18	0.15	0.10	**0.33**	0.15	0.08	0.10	0.10	0.05	0.05
YC-9*	0.10	0.13	**0.30**	0.13	**0.35**	0.15	0.05	0.05	0.08	0.13	0.13	0.08	0.08	0.00	0.03	0.00
YC-10	0.13	**0.20**	**0.48**	0.18	**0.23**	0.10	0.03	0.05	0.10	0.18	**0.25**	0.15	0.10	0.00	0.08	0.10
YC-12*	0.15	0.13	0.15	**0.20**	0.18	0.03	0.13	0.08	0.03	0.13	**0.25**	**0.28**	0.00	0.05	0.00	0.00
YC-13*	0.15	0.10	**0.20**	**0.20**	**0.23**	0.03	0.00	0.08	0.00	**0.20**	**0.20**	0.10	0.05	0.03	0.03	0.10
YC-18	0.13	0.08	**0.30**	0.18	**0.38**	0.13	0.05	0.18	0.05	0.15	**0.20**	**0.20**	0.08	0.08	0.03	0.10
YC-19	0.15	0.13	**0.35**	0.13	**0.28**	0.15	0.18	0.15	0.10	**0.33**	0.15	0.08	0.10	0.10	0.05	0.05

YC-21	0.13	0.10	**0.30**	**0.25**	**0.28**	0.18	0.15	0.18	0.08	**0.20**	**0.23**	**0.20**	0.03	0.03	0.08	0.00
YC-22	0.03	0.08	0.10	0.10	0.10	**0.25**	0.08	0.03	0.08	**0.23**	0.13	0.18	0.00	0.05	0.03	0.05
YC-24	0.13	0.10	**0.30**	0.03	**0.40**	**0.23**	0.10	0.18	0.08	**0.25**	0.10	0.15	0.08	0.08	0.05	0.08
YC-25	0.18	0.15	**0.33**	**0.20**	**0.38**	0.10	0.15	**0.23**	0.08	**0.25**	**0.23**	0.15	**0.20**	0.13	0.03	0.03
YC-27	0.08	0.05	**0.28**	0.08	**0.30**	0.18	0.15	0.10	0.00	0.15	**0.20**	**0.25**	0.08	0.00	0.00	0.10
YC-28	0.08	0.15	**0.30**	0.05	**0.30**	0.15	0.15	0.10	0.05	**0.25**	**0.28**	0.15	0.08	0.10	0.13	0.05
YC-29	**0.30**	0.15	**0.28**	0.10	**0.33**	0.10	0.13	0.13	0.08	0.10	0.15	0.15	0.05	0.10	0.03	0.10
YC-30*	0.15	0.18	**0.28**	0.08	**0.25**	0.15	0.03	0.10	0.08	**0.30**	0.18	0.10	0.03	0.05	0.00	0.03
YC-31	**0.20**	**0.25**	**0.50**	0.15	**0.40**	0.10	0.10	0.10	0.13	**0.35**	**0.20**	0.18	**0.20**	0.18	0.08	**0.23**
YC-32*	0.08	0.03	0.13	0.03	0.15	0.05	0.00	0.03	0.00	0.15	0.13	0.13	0.08	0.03	0.15	0.03
YC-33*	0.18	0.15	**0.30**	0.18	**0.33**	0.13	0.10	0.08	0.00	**0.30**	0.15	0.03	0.05	0.00	0.03	0.03
YN-1*	0.13	0.03	**0.30**	0.15	**0.28**	0.18	0.13	0.08	0.03	0.15	**0.20**	0.15	0.05	0.08	0.03	0.05
YN-2*	**0.20**	0.03	**0.43**	0.08	**0.23**	0.13	0.05	0.13	0.05	0.18	0.15	**0.20**	0.13	0.18	0.00	0.05
YN-3	0.08	0.05	0.15	0.05	**0.20**	**0.30**	0.05	**0.25**	0.13	**0.20**	0.15	0.18	0.08	0.10	0.03	0.05
YN-4*	0.08	0.03	**0.35**	0.15	**0.33**	0.18	0.03	0.03	0.05	**0.23**	**0.25**	0.13	0.08	0.08	0.00	0.13
YN-5	0.13	0.13	**0.33**	0.08	**0.40**	0.13	0.03	0.05	0.08	0.15	0.10	0.15	0.08	0.05	0.05	0.08
YN-6	0.13	**0.20**	**0.48**	**0.20**	**0.33**	**0.23**	0.05	0.05	0.08	**0.25**	0.15	0.15	0.13	0.13	0.05	0.10
YN-7	0.18	0.05	**0.30**	0.10	**0.38**	0.18	0.05	0.05	0.00	**0.28**	**0.20**	0.18	0.10	0.05	0.00	0.03
YN-8*	0.15	0.05	**0.28**	0.18	0.18	0.03	0.05	0.05	0.03	0.13	**0.20**	**0.40**	0.03	0.10	0.00	0.08
YN-10	0.08	0.10	**0.38**	0.05	**0.25**	0.10	0.08	0.08	0.03	**0.30**	**0.45**	**0.20**	0.08	0.05	0.00	0.03
YN-11	**0.20**	0.10	**0.33**	0.13	**0.38**	0.00	0.03	0.05	0.05	**0.35**	**0.23**	**0.23**	0.05	0.18	0.13	0.15
YN-12	**0.20**	0.15	**0.35**	**0.28**	**0.33**	0.15	0.13	0.18	0.13	**0.23**	0.10	**0.20**	0.15	0.08	0.03	0.05
YN-13	0.08	0.10	**0.28**	0.15	**0.28**	0.08	0.03	0.08	0.10	0.13	0.15	0.18	0.05	0.03	0.00	0.13
YN-15	0.15	0.10	**0.23**	0.03	0.03	0.08	0.03	0.10	0.03	0.15	0.05	**0.38**	0.05	0.15	0.00	0.00
YN-16	0.05	0.05	**0.30**	0.08	0.18	**0.23**	0.03	0.15	0.15	0.18	0.15	0.15	0.05	0.05	0.03	0.00
YN-17	0.10	0.05	**0.38**	0.15	**0.23**	0.10	0.08	0.10	0.08	0.15	0.10	0.13	0.03	0.05	0.05	0.05
YN-18	0.15	0.08	**0.33**	0.10	**0.25**	0.08	0.03	0.03	0.03	0.10	0.03	0.13	0.05	0.05	0.03	0.03

YN-19	0.13	0.08	**0.30**	**0.20**	0.18	0.08	0.13	0.10	0.05	**0.25**	**0.23**	**0.25**	0.08	0.05	0.00	0.03
YN-20	0.08	0.10	**0.35**	0.03	**0.28**	0.15	0.08	0.13	0.08	0.13	**0.20**	**0.20**	0.08	0.05	0.03	0.10
YN-21	0.15	0.05	**0.35**	0.13	**0.30**	0.18	0.05	0.08	0.05	0.15	0.08	**0.20**	0.03	0.10	0.05	0.10
YN-22	0.13	0.08	0.18	0.18	**0.20**	0.03	0.05	0.05	0.00	0.08	**0.25**	**0.40**	0.05	0.05	0.03	0.08
YN-23*	0.08	0.18	**0.35**	0.15	**0.30**	0.03	0.08	0.00	0.00	**0.23**	**0.25**	**0.33**	0.18	0.15	0.03	0.15
YN-24	0.15	0.08	**0.33**	0.03	0.13	**0.43**	0.05	**0.25**	0.15	**0.25**	0.13	0.08	0.03	0.10	0.03	0.08
YN-25*	0.03	0.03	**0.28**	0.18	**0.40**	**0.30**	0.10	0.18	0.15	0.05	**0.20**	0.15	0.10	0.03	0.03	0.03
QTX-3	0.08	0.10	**0.25**	0.13	**0.33**	0.05	0.05	0.08	0.03	**0.25**	**0.23**	0.08	0.08	0.13	0.13	0.08
QTX-4	0.03	0.10	**0.23**	0.08	0.15	0.03	0.08	0.05	0.05	0.18	0.18	**0.23**	0.10	0.10	0.10	0.10
QTX-5*	0.13	0.05	**0.20**	0.10	**0.23**	**0.23**	0.13	0.10	0.08	**0.28**	**0.20**	**0.20**	0.10	0.05	0.03	0.03
QTX-6	0.05	0.18	**0.20**	0.10	**0.28**	**0.13**	0.03	0.15	0.03	0.05	0.15	**0.20**	0.05	0.03	0.03	0.03
QTX-7*	0.15	0.15	**0.33**	0.18	**0.38**	0.05	0.00	0.08	0.05	0.18	0.13	0.18	0.08	0.15	0.15	0.08
QTX-9*	0.13	0.08	**0.25**	0.03	**0.23**	**0.23**	0.13	0.18	0.10	**0.35**	0.15	0.15	0.10	0.15	0.08	0.10
QTX-11	0.10	0.15	**0.40**	0.13	**0.23**	0.05	0.05	0.05	0.03	**0.28**	**0.30**	0.18	0.08	0.08	0.13	0.00
QTX13*	**0.25**	0.03	**0.25**	0.15	**0.28**	0.18	0.05	0.08	0.08	0.15	**0.20**	0.15	0.10	0.05	0.05	0.08
QTX-17	0.00	0.13	**0.25**	0.08	**0.28**	**0.20**	0.05	**0.23**	0.10	0.18	0.18	0.13	0.08	0.10	0.05	0.10
HSP-1*	0.05	0.08	**0.23**	0.00	0.13	0.00	0.13	0.10	0.05	**0.40**	0.10	**0.23**	**0.38**	0.05	0.03	0.08
HSP-2*	0.10	0.08	**0.23**	0.05	0.10	0.03	0.03	0.05	0.05	**0.25**	0.13	0.18	0.08	0.05	0.05	0.05
HSP-3*	**0.20**	0.08	**0.35**	0.08	**0.23**	0.13	0.05	0.10	0.15	**0.25**	0.18	0.18	0.18	0.05	0.08	**0.20**

*The representative wines of each sub-region were selected and evaluated for QDA. [a] The attributes with significant differences other than aged attributes were used as the standard for selecting representative wines of each sub-regio

Table S5 Basic information of Cabernet Sauvignon dry red wines from six sub-regions at the Eastern Foothills of Helan Mountain in Ningxia

Years	SZS	HL	XX	YN	QTX	HSP	Numbers
2015	1	0	4	1	1	0	7
2016	0	1	4	3	1	1	10
2017	0	2	5	8	2	0	17
2018	0	2	5	6	3	1	17
2019	1	4	3	4	1	1	14
2020	0	1	2	1	1	0	5
2021	0	1	0	0	0	0	1
Total	2	11	23	23	9	3	71

Table S6. Physicochemical indexes and information of Cabernet Sauvignon dry red wine in six sub-regions at the Eastern foothills of Helan Mountain in Ningxia

Samples	Vintages	Regions	Alcohol (%vol)	Total acidity (g/L)	Volatile acidity (g/L)	Total sugar (g/L)	pH
SZS-1	2015	SZS	14.65±0.00	5.50±0.00	0.87±0.00	3.80±0.00	4.04±0.00
SZS-2	2019	SZS	14.48±0.00	5.70±0.00	0.76±0.00	3.07±0.06	3.81±0.00
HL-1	2016	HL	15.08±0.00	5.70±0.00	0.81±0.01	2.47±0.06	3.99±0.01
HL-2*	2017	HL	14.29±0.00	5.70±0.00	0.65±0.00	1.83±0.06	3.95±0.00
HL-3	2017	HL	15.16±0.01	5.50±0.00	0.61±0.00	2.93±0.06	3.90±0.01
HL-4*	2020	HL	16.74±0.01	5.53±0.06	0.65±0.00	3.27±0.06	4.04±0.00
HL-5*	2021	HL	16.49±0.00	7.60±0.00	0.53±0.01	10.23±0.06	3.82±0.00
HL-6*	2019	HL	15.58±0.00	5.90±0.00	0.67±0.01	3.90±0.10	3.76±0.00
HL-7	2019	HL	15.40±0.00	5.80±0.00	0.46±0.00	3.90±0.00	3.77±0.00
HL-8	2018	HL	15.53±0.01	6.00±0.00	0.55±0.01	5.87±0.06	3.79±0.00
HL-9	2019	HL	15.34±0.01	6.20±0.00	0.70±0.01	3.80±0.00	3.81±0.01
HL-10*	2019	HL	14.32±0.00	6.13±0.06	0.60±0.01	4.50±0.00	3.71±0.00
HL-11	2018	HL	15.08±0.01	5.40±0.00	0.71±0.01	4.33±0.06	4.02±0.00
YC-1	2018	XX	16.13±0.01	6.40±0.00	0.64±0.00	3.73±0.06	3.78±0.00
YC-2*	2016	XX	14.66±0.01	5.00±0.00	0.67±0.01	3.00±0.00	3.80±0.00
YC-3*	2015	XX	14.10±0.01	5.10±0.00	0.60±0.00	2.80±0.00	3.71±0.00
YC-4	2016	XX	13.99±0.00	5.00±0.00	0.66±0.01	2.60±0.00	3.90±0.00
YC-5	2017	XX	14.14±0.01	4.90±0.00	0.72±0.00	2.87±0.06	3.99±0.00
YC-7*	2019	XX	14.70±0.01	6.00±0.00	0.86±0.01	2.63±0.06	3.92±0.00
YC-9*	2018	XX	13.79±0.01	5.40±0.00	0.75±0.00	2.47±0.06	3.92±0.00
YC-10	2015	XX	15.09±0.00	5.90±0.00	0.79±0.00	3.77±0.06	3.81±0.01
YC-12*	2019	XX	15.59±0.01	7.57±0.06	0.69±0.00	3.93±0.06	3.54±0.00
YC-13*	2020	XX	15.42±0.01	5.50±0.00	0.59±0.01	3.10±0.1.	3.99±0.00
YC-18	2020	XX	14.43±0.00	5.40±0.00	0.74±0.01	2.90±0.00	4.05±0.01
YC-19	2017	XX	14.16±0.01	5.17±0.06	0.61±0.00	3.03±0.06	3.84±0.01
YC-21	2016	XX	14.62±0.01	5.80±0.00	0.67±0.00	2.87±0.06	3.80±0.00
YC-22	2015	XX	14.52±0.00	5.40±0.00	0.57±0.01	2.93±0.06	3.89±0.00
YC-24	2017	XX	14.71±0.00	5.60±0.00	0.56±0.00	3.30±0.00	3.78±0.00
YC-25	2016	XX	15.47±0.00	5.57±0.06	0.58±0.01	3.37±0.06	3.82±0.01
YC-27	2018	XX	15.68±0.00	6.00±0.00	0.70±0.01	4.13±0.06	3.81±0.00
YC-28	2017	XX	15.05±0.01	5.80±0.00	0.75±0.00	5.13±0.06	3.98±0.01
YC-29	2018	XX	14.46±0.00	6.50±0.00	0.66±0.01	3.53±0.06	3.62±0.00
YC-30*	2015	XX	15.37±0.01	6.00±0.00	1.02±0.00	3.40±0.00	3.88±0.00
YC-31	2018	XX	14.81±0.00	6.13±0.06	0.81±0.01	2.10±0.00	3.76±0.00
YC-32*	2019	XX	15.22±0.00	6.30±0.00	0.76±0.01	5.23±0.06	3.72±0.01
YC-33*	2017	XX	14.61±0.01	5.00±0.00	0.74±0.00	2.53±0.06	4.01±0.00
YN-1*	2019	YN	15.38±0.01	5.60±0.00	0.72±0.00	3.93±0.06	3.76±0.01

YN-2*	2018	YN	14.73±0.01	5.10±0.00	0.69±0.01	4.00±0.00	3.79±0.00
YN-3	2017	YN	15.14±0.00	5.30±0.00	0.74±0.00	3.70±0.00	3.73±0.00
YN-4*	2017	YN	14.95±0.00	5.13±0.06	0.76±0.01	3.70±0.1.	3.84±0.00
YN-5	2018	YN	14.22±0.01	5.50±0.00	0.60±0.01	2.97±0.06	3.73±0.00
YN-6	2016	YN	14.17±0.00	5.70±0.00	0.49±0.01	3.90±0.00	3.62±0.00
YN-7	2017	YN	14.02±0.00	5.60±0.00	0.53±0.00	4.03±0.06	3.68±0.00
YN-8*	2017	YN	13.81±0.00	5.10±0.00	0.53±0.00	2.97±0.06	3.65±0.00
YN-10	2017	YN	13.97±0.01	5.30±0.00	0.69±0.00	3.20±0.00	3.69±0.00
YN-11	2018	YN	13.89±0.01	5.50±0.00	0.69±0.00	3.23±0.06	3.69±0.00
YN-12	2019	YN	13.14±0.00	5.30±0.00	0.58±0.00	3.03±0.06	3.63±0.00
YN-13	2016	YN	14.63±0.00	5.50±0.00	0.64±0.00	3.23±0.15	3.72±0.00
YN-15	2017	YN	15.54±0.01	5.80±0.00	0.85±0.01	2.30±0.00	4.10±0.00
YN-16	2019	YN	14.1±0.01	6.00±0.00	0.53±0.01	3.07±0.06	3.72±0.00
YN-17	2020	YN	13.36±0.00	5.40±0.00	0.59±0.01	3.10±0.00	3.68±0.00
YN-18	2017	YN	13.5±0.01	5.60±0.00	0.60±0.01	3.00±0.10	3.60±0.00
YN-19	2018	YN	13.35±0.01	5.20±0.00	0.66±0.01	1.73±0.06	3.77±0.00
YN-20	2015	YN	15.62±0.01	6.20±0.00	0.69±0.00	3.47±0.06	3.73±0.00
YN-21	2016	YN	15.56±0.00	6.00±0.00	0.62±0.00	3.70±0.00	3.83±0.00
YN-22	2017	YN	14.83±0.01	5.27±0.06	0.78±0.01	2.10±0.10	4.23±0.00
YN-23*	2018	YN	14.74±0.01	5.00±0.00	0.72±0.01	4.43±0.06	4.12±0.00
YN-24	2018	YN	14.96±0.01	5.57±0.06	0.86±0.01	2.73±0.06	3.96±0.00
YN-25*	2019	YN	15.19±0.01	6.90±0.00	0.72±0.01	3.77±0.06	3.58±0.00
QTX-3	2019	QTX	14.77±0.00	5.30±0.00	0.75±0.00	3.93±0.05	4.01±0.00
QTX-4	2020	QTX	13.83±0.01	5.10±0.00	0.50±0.00	2.97±0.05	3.85±0.00
QTX-5*	2017	QTX	15.40±0.00	5.70±0.00	0.70±0.01	1.83±0.06	4.13±0.00
QTX-6	2018	QTX	13.53±0.01	5.80±0.00	0.60±0.00	2.20±0.10	3.81±0.01
QTX-7*	2016	QTX	15.13±0.01	5.60±0.00	0.72±0.01	3.90±0.00	3.81±0.00
QTX-9*	2017	QTX	14.23±0.00	5.30±0.00	0.70±0.01	2.27±0.06	3.98±0.00
QTX-11	2018	QTX	14.99±0.01	6.20±0.00	0.90±0.01	2.90±0.00	3.93±0.00
QTX-13*	2015	QTX	13.81±0.00	5.60±0.00	0.58±0.00	3.80±0.00	3.76±0.00
QTX-17	2018	QTX	14.22±0.00	5.80±0.00	0.59±0.01	3.20±0.10	3.63±0.00
HSP-1*	2018	HSP	13.29±0.01	5.30±0.00	0.56±0.01	4.90±0.00	3.70±0.00
HSP-2*	2019	HSP	14.36±0.01	6.60±0.00	0.57±0.01	4.50±0.00	3.58±0.01
HSP-3*	2016	HSP	14.87±0.01	5.70±0.00	0.56±0.00	3.30±0.10	3.76±0.01

* The representative wines of each sub-region were selected and evaluated for QDA.

Table S7. Preparation of reference standards for each sensory attribute *

NO	Attributes	References
1	Blackberry	Dissolve 20 μL of blackberry essence in 20 mL of simulated wine solution.
2	Vanilla	Dissolve 20 μL of vanilla essence in 20 mL of simulated wine solution.
3	Cherry	Dissolve 20 μL of cherry essence in 20 mL of simulated wine solution.
4	Smoke	Smoky wines tasted by experts.
5	Violet	Le nez du vin
6	Raspberry	0.35 g of raspberry was treated with liquid nitrogen, and the crumbs were dissolved in 20 mL of simulated wine.
7	Herbaceous	0.35 g of herbaceous was treated with liquid nitrogen, and the crumbs were dissolved in 20mL of simulated wine.
8	Licorice	Dissolve 20 μL of licorice essence in 20 mL of simulated wine solution.
9	Green pepper	0.35 g of green pepper was treated with liquid nitrogen, and the crumbs were dissolved in 20mL of simulated wine.
10	Caramel/Taffy	Le nez du vin
11	Mint	Dissolve 20 μL of mint essence in 20 mL of simulated wine solution.

12	Jam	Strawberry jam
13	Hawthorn	0.35 g of hawthorn was treated with liquid nitrogen, and the crumbs were dissolved in 20 mL of simulated wine.
14	Black pepper	Physical purchases from supermarkets.
15	Animal	Animal wines tasted by experts.
16	Coffee	Physical purchases from supermarkets.
17	Cream	Dissolve 20 μL of cream essence in 20 mL of simulated wine solution.
18	Grass	0.35 g of grass was treated with liquid nitrogen, and the crumbs were dissolved in 20 mL of simulated wine.
19	Spices	Cinnamon, cloves bought from the supermarket
20	Strawberry	Dissolve 20 μL of strawberry essence in 20 mL of simulated wine solution.
21	Chocolate	Dissolve 20 μL of chocolate essence in 20 mL of simulated wine solution.
22	Mulberry	0.35 g of mulberry was treated with liquid nitrogen, and the crumbs were dissolved in 20 mL of simulated wine.
23	Coconut	Coconut milk
24	Cinnamon	Physical purchases from supermarkets.

25	Green	2.5 µL methoxypyrazine mixed standard (8 ng/L) dissolved in 20 mL simulated wine.
26	Oak	Oak chips
27	Toast	Roasted chestnuts
28	Leather	Sheepskin products
29	Sophora flower	Dissolve 20 µL of sophora flower perfume in 20 mL of simulated wine solution.
30	Rose	Dissolve 20 µL of rose essence in 20 mL of simulated wine solution.
31	Toasted hazelnuts/toast	Le nez du vin
32	Honey	Dissolve 20 µL of honey essence in 20 mL of simulated wine solution.
33	Baked sweet potatoes	Dissolve 20 µL of baked sweet potatoes essence in 20 mL of simulated wine solution.
34	Alcohol	Simulated wine solution.
35	Apple/Pear	Dissolve 20 µL of apple and pear essence in 20 mL of simulated wine solution.
36	Banana	Dissolve 20 µL of banana essence in 20 mL of simulated wine solution.
37	Peach/Apricot	Dissolve 20 µL of blue berry essence in 20 mL of simulated wine solution.

38	Forest floor	Soil after rain
39	Plum	0.35 g of plum was treated with liquid nitrogen, and the crumbs were dissolved in 20 mL of simulated wine.
40	Prunes	0.35 g of prunes was treated with liquid nitrogen, and the crumbs were dissolved in 20 mL of simulated wine.
41	Blueberry	Dissolve 20 μL of blue berry essence in 20 mL of simulated wine solution.
42	Dried/Preserved Fruit	Raisin
43	Hay	0.35 g of hay was treated with liquid nitrogen, and the crumbs were dissolved in 20 mL of simulated wine.
44	Clove	Physical purchases from supermarkets.
45	Black currant	Le nez du vin.
46	Mushroom	Physical purchases from supermarkets.
47	Mineral	Flintstones
48	Cedar	0.35 g of cedar was treated with liquid nitrogen, and the crumbs were dissolved in 20 mL of simulated wine.
49	Red currant	Le nez du vin.

* The model solution was prepared using12% ethanol/water solution, pH = 3
